# PReS-FINAL-2142: Predictors of persistent remission following etanercept (ETN) withdrawal in patients with juvenile idiopathic arthritis (JIA)

**DOI:** 10.1186/1546-0096-11-S2-P154

**Published:** 2013-12-05

**Authors:** F Perfetti, F Gesualdo, V Messia, A Insalaco, C Bracaglia, M Pardeo, R Nicolai, L Meli, F De Benedetti, S Magni Manzoni

**Affiliations:** 1 Pediatric Rheumatology Unit, Rome, Italy; 2 Epidemiology Unit, IRCCS Bambino Gesù Children's Hospital, Rome, Italy

## Introduction

At present, no guidelines on the appropriate withdrawal of ETN in JIA patients in clinical remission exist. The identification of predictors of clinical remission off medication (CROM) in these patients represents the initial step in their development.

## Objectives

To evaluate the rate of CROM in JIA patients after ETN withdrawal; to identify predictors of CROM in these patients.

## Methods

Retrospective data of polyarticular JIA patients who discontinued ETN due to clinical remission on therapy and had a follow-up period after ETN withdrawal of at least 12 months were collected, including: age, number and type of active joints at disease onset, sex, ANA status, disease duration before ETN start, number and type of active joints, ESR, and CRP at the time of ETN start, duration of clinical remission on medication and off-medication. Based on their clinical course after ETN withdrawal, patients were divided into 2 groups: 1) with CROM 2) with disease flare. Demographic, clinical and laboratory features were compared between the two groups.

## Results

A total of 49 patients were included in the study. 11 patients (22,4%) achieved CROM, while 77.6% experienced a disease flare after a median of 4,8 months (IQ 2.9-9.2). Patients with CROM showed a significantly lower frequency of ANA positivity, ESR values < 20 mm/1 h and ankle involvement at the beginning of ETN treatment than those who relapsed (respectively, *p 0.034, 0.044 and 0.008*). None of the other parameters showed any difference in the two groups.

## Conclusion

Only 1/5 of JIA patients in our cohort achieved CROM. Children with ANA negativity, ESR < 20 mm/1 h and without ankle involvement have had a greater likelihood of achieving CROM. Further prospective studies with longer follow-up are needed to confirm these results.

## Disclosure of interest

None declared.

